# A New Piece of the *Shigella* Pathogenicity Puzzle: Spermidine Accumulationby Silencing of the *speG* Gene

**DOI:** 10.1371/journal.pone.0027226

**Published:** 2011-11-10

**Authors:** Marialuisa Barbagallo, Maria Letizia Di Martino, Lucia Marcocci, Paola Pietrangeli, Elena De Carolis, Mariassunta Casalino, Bianca Colonna, Gianni Prosseda

**Affiliations:** 1 Department of Biology and Biotechnology “C.Darwin”, Institut Pasteur-Fondazione Cenci Bolognetti, University of “Sapienza” Rome, Rome, Italy; 2 Department of Biochemical Sciences “A. Rossi Fanelli”, University “Sapienza” of Rome, Rome, Italy; 3 Istituto di Microbiologia, Università Cattolica del Sacro Cuore, Rome, Italy; 4 Department of Biology, University of Roma, Rome, Italy; Monash University, Australia

## Abstract

The genome of *Shigella*, a gram negative bacterium which is the causative agent of bacillary dysentery, shares strong homologies with that of its commensal ancestor, *Escherichia coli*. The acquisition, by lateral gene transfer, of a large plasmid carrying virulence determinants has been a crucial event in the evolution towards the pathogenic lifestyle and has been paralleled by the occurrence of mutations affecting genes, which negatively interfere with the expression of virulence factors. In this context, we have analysed to what extent the presence of the plasmid-encoded *virF* gene, the major activator of the *Shigella* regulon for invasive phenotype, has modified the transcriptional profile of *E. coli*. Combining results from transcriptome assays and comparative genome analyses we show that in *E. coli* VirF, besides being able to up-regulate several chromosomal genes, which potentially influence bacterial fitness within the host, also activates genes which have been lost by *Shigella*. We have focused our attention on the *speG* gene, which encodes spermidine acetyltransferase, an enzyme catalysing the conversion of spermidine into the physiologically inert acetylspermidine, since recent evidence stresses the involvement of polyamines in microbial pathogenesis. Through identification of diverse mutations, which prevent expression of a functional SpeG protein, we show that the *speG* gene has been silenced by convergent evolution and that its inactivation causes the marked increase of intracellular spermidine in all *Shigella* spp. This enhances the survival of *Shigella* under oxidative stress and allows it to better face the adverse conditions it encounters inside macrophage. This is supported by the outcome of infection assays performed in mouse peritoneal macrophages and of a competitive-infection assay on J774 macrophage cell culture. Our observations fully support the pathoadaptive nature of *speG* inactivation in *Shigella* and reveal that the accumulation of spermidine is a key determinant in the pathogenicity strategy adopted by this microrganism.

## Introduction

Polyamines are ubiquitous, small polycationic compounds associated with a variety of biological processes: protein translation, gene regulation, stress resistance and differentiation [1], [2]. Major representatives of this class of molecule are putrescine, cadaverine, spermidine and spermine.

**Figure 1:**
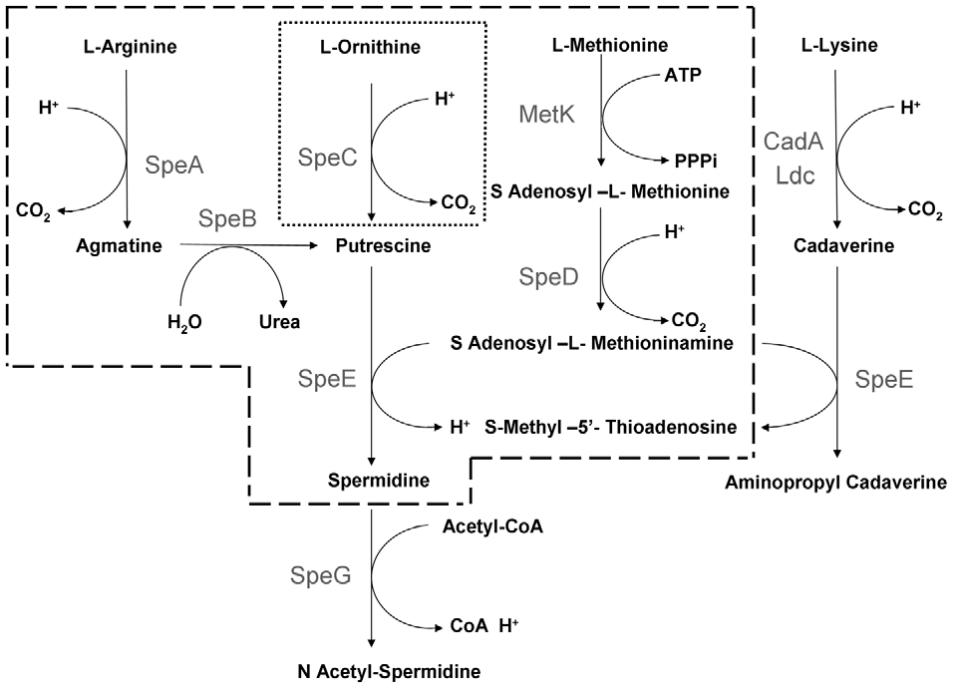
Superpathway of polyamine biosynthesis I in *E. coli* and *Shigella* spp. Schematic diagram depicting the pathway of polyamine biosynthesis I in *E. coli*. Steps bounded by the dashed lines are conserved in *Shigella spp*. The step enclosed by dotted lines is absent in *S. boydii*. Data were drawn according to http://ecocyc.org.

In bacteria, the global level of polyamines is regulated on the one hand by collective effects of catabolism and efflux mechanisms and, on the other, by biosynthetic pathways and uptake mechanisms [2], [3]. Figure 1 reports the superpathway of polyamine biosynthesis I in *Escherichia coli* (from http:ecocyc.org database), which is able, like most γ-proteobacteria, to synthesize cadaverine, putrescine and spermidine, but not spermine [2], [4]. Cadaverine is produced through the combined action of an inducible and a constitutive lysine decarboxylase, encoded respectively by the *cadA* and *ldc* genes [5], [6]. It is then converted to aminopropylcadaverine by the SpeE protein. Putrescine results from direct ornithine decarboxylation, mediated by the SpeC decarboxylase, and from arginine decarboxylation followed by agmatine ureohydrolization determined by the SpeA and SpeB proteins, respectively. Spermidine originates from the condensation of putrescine with decarboxylated S-adenosylmethionine, performed by the SpeE [2], [7]. High levels of spermidine are toxic for *E. coli* cells, but spermidine acetylation, catalysed by SpeG, inactivates the polyamine. Acetylspermidine is thought to be either stored by the cells or secreted [8].

During recent years, strong evidence has accumulated on the role of polyamines in microbial pathogenesis. In *Pseudomonas aeruginosa*, the deletion of genes involved in spermidine uptake significantly decreases the expression of Type III Secretion Systems (TTSS) [9]. In *Streptococcus pneumoniae*, polyamine biosynthesis and transport mechanisms are intricately linked to the fitness, survival and pathogenesis of this pathogen in host microenvironments [10]. In *Yersinia pestis* and in *Vibrio cholerae*, polyamines have been implicated in the control of biofilm formation [11], [12]. In *Proteus mirabilis*, the inactivation of the *speAB* genes, involved in putrescine biosynthesis, leads to the loss of the swarming phenotype [13] linked to the expression of some virulence genes [14]. The fungal pathogen *Pneumocystis jirovecii* produces high levels of spermidine, N^1^-acetylspermine and N^1^-acetylspermidine, thus inducing apoptosis of alveolar macrophages [15].

We have focused our analysis on spermidine metabolism in *Shigella*, a facultative intracellular pathogen causing a severe enteric syndrome in humans, mainly in the developing world. Shigellosis is extremely contagious and, although usually self-limiting, may be fatal in children [16]. The highly sophisticated infectious strategy of *Shigella* banks on the capacity of this pathogen to invade, disrupt, and cause inflammatory destruction of the intestinal epithelial barrier. Once ingested, *Shigella* moves directly down to the colon where it gains access to the intestinal mucosa by invading specialized epithelial cells, the M cells in Peyer's patches, and subsequently infecting adjacent cells in intestinal crypts. Once the bacteria reach the lymphoid follicles, they encounter resident macrophages, where they multiply, induce apoptosis and give rise to an inflammatory response, the hallmark of this enteric disease. This, in turn, induces transmigration of polymorphonucleated leukocytes (PMN) through the tight junctions between epithelial cells. As PMNs begin to migrate, bacteria released from killed macrophages can invade the epithelial monolayer, accessing the basolateral surfaces of the colonic epithelium. Bacterial entry into the host cells is induced by the TTSS-secreted Ipa proteins, which activate host signaling pathways and induce a focused reorganization of the cytoskeletal actin around the bacterial cell. Inside the host cell, *Shigella* disrupts the vacuole membrane and escapes into the cytoplasm, where it multiplies, and moves by inducing local actin polymerization at one pole of the bacterium. The actin-based motility propels *Shigella* through the cytoplasm and facilitates intercellular dissemination towards the neighboring cells [17], [18].

The cellular pathogenesis and clinical presentation of shigellosis are the sum of the complex action of a large number of bacterial virulence factors mainly located on a large virulence plasmid (pINV) [19]. The availability of complete sequenced genomes of several *Shigella* strains has given new insight about the molecular evolution of this bacterial pathogen from its ancestor, the commensal *E. coli* [20]. While the acquisition of pINV is regarded as one of the most critical events in the evolution of *Shigella* towards a pathogenic lifestyle, a significant complementary step has been the emergence of so-called pathoadaptive mutations [21]. This has led to the inactivation of several chromosomal genes, which negatively interfere with the expression of virulence factors required for the survival within the host [22], [23]. In particular, the silencing of the *cad* genes, involved in the synthesis of a specific polyamine, cadaverine, appears crucial for the optimization of the pathogenicity process in *Shigella* [22], [23]. Cadaverine negatively interferes with *Shigella*-induced pro-inflammatory events by inhibiting PMN migration to the infection loci [24] and may stabilize the endosomal membrane, hindering the release of *Shigella* cells into the cytoplasm of infected cells [25].

In this study, we show, by convergent evolution, that *Shigella* has lost another crucial gene involved in polyamine metabolism, *speG*. The loss of this gene, which encodes spermidine acetyltransferase, allows for higher concentrations of endogenous spermidine. We also show that restoring SpeG activity confers upon *Shigella* a higher sensitivity to oxidative stress and reduces bacterial survival inside macrophages. This strongly supports the hypothesis that *speG* inactivation constitutes a previously unrecognized patho-adaptative mutation common to all member of *Shigella* genus.

## Results

### *speG* expression depends on the VirF regulatory protein

The *Shigella* pINV plasmid contains, besides genes involved in the invasive process, positive activators necessary for the induction of host cell colonization. Among them, a critical role is played by the transcriptional regulator VirF. It is encoded by a gene activated in response to host temperature [26] and is located outside the large pathogenicity island carrying most virulence determinants [19]. VirF triggers a cascade of events: it activates the transcription of the gene coding for the secondary regulator, VirB, which activates several operons coding for the invasion genes [27].

To understand whether the arrival of VirF by acquisition of pINV might have altered the transcriptional program of the ancestor *E. coli* and promoted the inactivation of genes potentially detrimental to the full expression of the invasive phenotype, we performed a global transcriptional analysis of *E. coli* cells expressing or lacking the *virF* gene. To this end we set up a microarray experiment using the *E. coli* K12 MG1655 strain [28], carrying the *virF*-encoding plasmid pMYSH6504 [29] or its *virF*-depleted derivative pMY6504R (Table S1). This analysis was performed on an *E. coli* K12-V2 array (MWG) containing 4288 gene-specific oligonucleotide probes representing the complete *E. coli* (K12) genome. This experiment revealed the presence of several *E. coli* genes activated at least two fold by VirF, either directly or indirectly (Table S2). Comparative genome analysis with *Shigella* reveals that these genes can be subdivided into two groups: genes which are common to *Shigella* and *E. coli*, and genes that are deleted or inactivated in *Shigella*. Interestingly in the first group we have identified several, highly induced, genes coding heat shock proteins including *ibpA*, *htpG*, *GroL/GroS*, *dnaK* and *lon* (Table S2).

The existence of the second group suggests that some VirF-activated genes might have exerted a perturbing effect on the *Shigella* invasive process, thus becoming silenced during evolution optimizing bacterial survival in the host. Most of the VirF-activated genes silenced in *Shigella* are poorly characterized. An exception is represented by *speG*, which encodes spermidine acetyltransferase (SAT) (Table S2). The *speG* gene belongs to the *ynfB-speG* operon. While no function has been yet attributed to the *ynfB* gene in *E. coli*, SAT prevents spermidine accumulation, and the consequent toxic effects, by modifying spermidine to an inert form [8].

**Figure 2:**
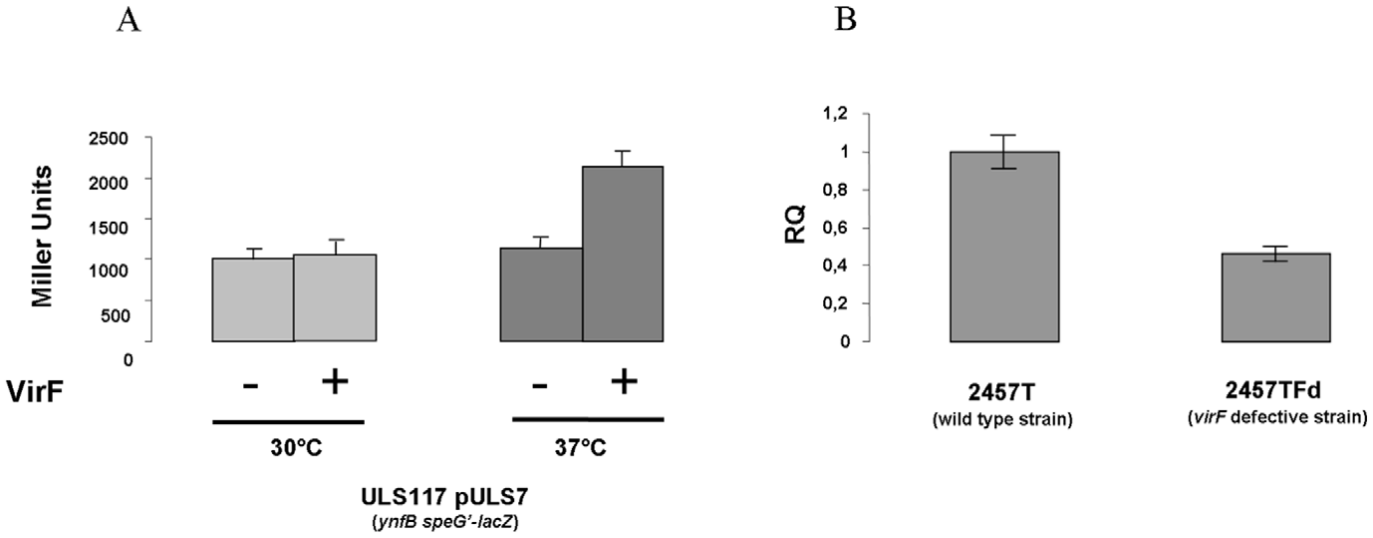
VirF positively controls the *ynfB-speG* operon. **A.** β-galactosidase activity of the *SpeG-LacZ* fusion carried by plasmid pULS7 was determined in *E. coli* ULS153 in the presence of pMYSH6504, a plasmid containing a functional *S. flexneri virF* gene, or of its *virF*-depleted variant pMY6504R. Cells were grown at 30°C or 37°C in LB medium and assayed for β-galactosidase at OD_600_ 0.5–0.6. The values reported are expressed in Miller Units and represent the average ± standard deviation of at least 3 independent experiments. **B.** The *in vivo ynfB-speG* transcription was monitored by real-time PCR in *S. flexneri* 2457T and its *virF* defective derivative 2457TFd. Strains were grown at 37°C in LB medium. At least three wells were run for each sample and the error bars display the calculated maximum (RQMax) and minimum (RQMin) expression levels that represent standard error of the mean expression level (RQ value).

To confirm *speG* activation by VirF, we analysed the activity of the *ynfB-speG* operon by constructing a translational P*_ynfB speG_*- *lacZ* fusion reporter plasmid (pULS7). The β-galactosidase assay (Fig. 2A) performed on strain ULS153 pULS7, in the presence or in the absence of a *virF*-containing plasmid (pMYSH6520 or pMY6520R), confirms that the expression of *speG* is induced by VirF. The induction is observed only at 37°C, as expected considering the thermodependency of *virF* expression [26]. A further confirmation of the role played by VirF on *speG* induction has been obtained in a *Shigella* background by monitoring *speG* transcription in a real-time PCR assay. This was performed using *S. flexneri* strain 2457T (which harbours a frameshift mutation in the *speG* gene inducing the synthesis of a truncated SAT protein without altering the transcriptional activity of the *ynfB-speG* operon) and its *virF*-deleted derivative 2457TFd. As reported in Fig. 2B, the lack of a functional *virF* gene in strain 2457Fd is paralleled by a two-fold reduction of *speG* expression, thus confirming the results obtained in the *E. coli* background.

These results indicate that VirF is able to interfere with the regulation of several genes present on the *E. coli* chromosome and that this may, in turn, promote the inactivation of genes potentially detrimental to the full expression of the invasive phenotype.

### Molecular characterization of the *speG* locus in *Shigella*

**Figure 3:**
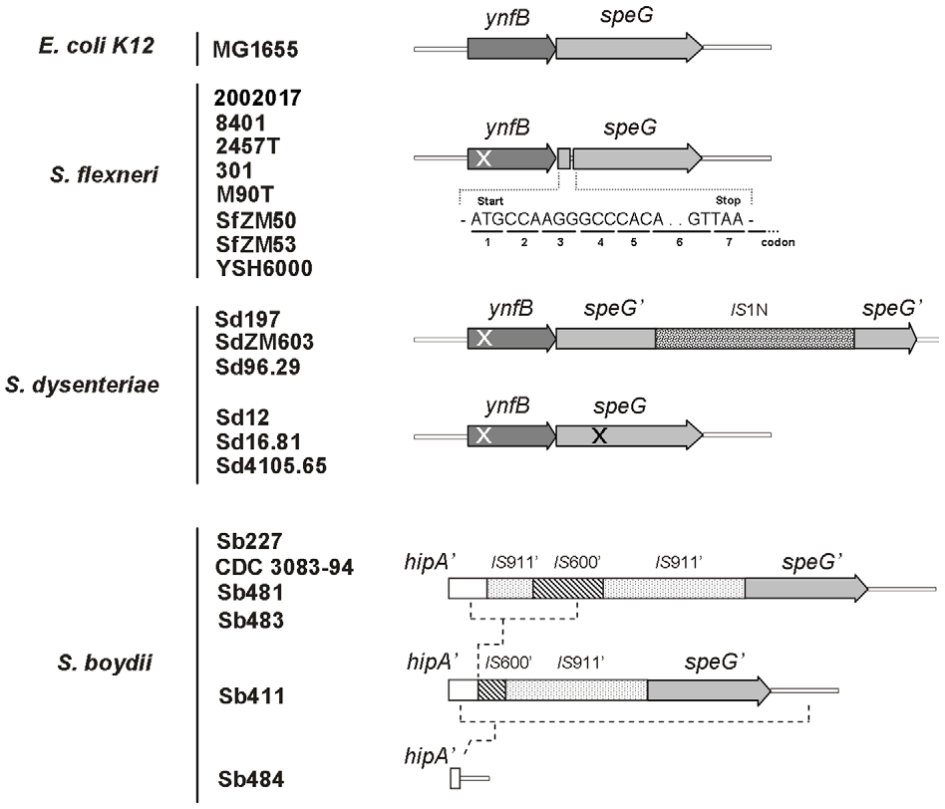
Inactivation of the *ynfB-speG* locus in *Shigella spp.* has been attained by convergent evolution. The operon on the top is based on the *E. coli K12* MG1655 sequenced (http://www.ncbi.nlm.nih.gov/genome). Arrows indicate the orientation of *ynfB* and *speG* genes. Point mutation, leading to the Il4P substitution, within the *ynfB* gene found in most *S. flexneri* and *S. dysenteriae* strains analysed is indicated by a white cross. Point mutation, leading to the S56R substitution, detected in some *S. dysenteriae* strains is indicated by a black cross. All the *S. flexneri* strains show the interruption of SpeG coding sequence due to a stop codon (7^th^) resulting from a dinucleotide (GT) deletion indicated by two full stops. The comparative analysis of *speG* sequences from *S. boydii* strains shows three different structures that may result from at least two deletion steps, which are schematized by dashed lines. Finally, the *ynfB-speG* locus of *S. sonnei* is not reported since it has been completely lost.

An *in silico* analysis, performed on genome sequences currently available on public databases (http://www.mgc.ac.cn/ShiBASE/ and http://www.ncbi.nlm.nih.gov/), highlights that *speG* is always defective in *Shigella* and that its inactivation has been obtained by diverse strategies. To verify the widespread nature of *speG* inactivation in *Shigella* and analyse the molecular rearrangements that might have led to *speG* silencing, we sequenced the *speG* locus of a large collection of *Shigella* strains (*S. flexneri*, *S. boydii*, *S. dysenteriae* and *S. sonnei*) isolated over several years in different geographic areas (Table S1). The results are reported in Fig. 3.

The *in silico* approach indicates that, in *S. flexneri* strains 2002017, 8401, 2457T and 301, *speG* inactivation is due to a dinucleotide deletion, which produces a TAA stop codon in the initial part of the coding sequence (see Fig. 3). The presence of the same mutation was confirmed in all *S. flexneri* strains but two: namely SfZM49 and SfZM43 [30]. Southern analysis revealed that remnants of the *speG* locus are actually present in SfZM49, while in SfZM43 the *speG*-containing region is completely lost (data not shown). Interestingly, SfZM43 belongs to serotype 6, previously considered phylogenetically distant from all other *S. flexneri* serotypes [20]. Moreover, in all *Shigella* strains analysed we found a non-synonymous mutation in the *ynfB* gene, responsible for an I14P amino acid substitution (JF737027, JF737028, JF737029, JF737030).

In *S. dysenteriae*, loss of *speG* functionality has been attained through two diverse strategies. Two strains, SdZM603 and Sd96.29 (both of serotype 1A), harbour an *IS*1N insertion (www-is.biotoul.fr and JF742750, JF742751) within the *speG* gene, as previously observed for sequenced strain Sd197. The overall genetic organization of strains Sd12, Sd16.81 and Sd4105.65 (all of serotype 2A) is identical to that of *E. coli* K12, but sequence analysis reveals that these *S. dysenteriae* strains share several point mutations, out of which only one gives rise to a non-synonymous mutation determining a S56R substitution in the SpeG protein sequence (JF737021, JF737025, JF737026). All *S. dysenteriae* strains analysed share the same non-synonymous mutation found in the *ynfB* gene of *S. flexneri* strains. Finally, we observe that no relevant mutations are located in the *ynfB-speG* promoter nor in the intergenic region. To check whether the S56R non-synonymous mutation could account for the synthesis of a defective protein, we cloned a fragment containing the entire *ynfB-speG* operon of *S. dysenteriae* Sd12 and *E. coli* MG1655 into the pGEM-T easy vector, thus obtaining plasmids pULS12 and, respectively, pULS11. We then compared the polyamine patterns of an *E. coli speG* defective strain (ULS117) complemented with pULS12 or with pULS11. As opposed to pULS11, the introduction of pULS12 does not restore production of acetylspermidine (Table 1), confirming that the *ynfB-speG* operon of *S. dysenteriae* Sd12 encodes an inactive SpeG protein.

**Table 1:** Comparative polyamine content of *E. coli speG* defective strain complemented with *speG* from *S. dysenteriae*.

polyamines	ULS117	ULS117 pULS12	ULS117 pULS11	MG1655
N-SPD	n.d.	n.d.	55±4.1	48.0±3.5
PUT	232.0±9.7	144.50±5.6	160±7.5	184.9±7.2
CAD	7.5±0.5	4.2±0.5	6.3±0.9	8.1±0.9
SPD	37.7±4.1	21.8±1.7	12±1.5	14.3±1.2

Values reported are in nmol per mg of total proteins and represent the average ± standard deviations from of triplicate determinations (n.d. = not detected) N-SPD: Acetylspermidine; PUT: Putrescine; CAD: Cadaverine; SPD: spermidine.

The analysis of the *S. boydii* Sb227 and CDC 3083-94 sequenced genomes reveals that *speG* has been inactivated by the insertion of an *IS*911 element, which carries, within its sequence, another *IS* element (IS600). A short deletion is present at the *IS*600-*IS*911 junction. Among the *S. boydii* strains analysed only two, Sb481 and Sb483, carry a *speG* locus with the same genetic organization as strains Sb227 and CDC 3083-94 (JF737022, JF737024). In particular, with the exception of strains Sb51 and Sb485, which harbour a complete deletion of *speG* locus, in the remaining strains we observe a progressive reduction of the Sb227 *speG*-*IS* arrangement: strain Sb411 shows a 365 bp deletion starting from within the *hipA* sequence and ending within the *IS*600 sequence (JF737020), while strain Sb484 (JF737023) carries a 1446 bp deletion extending from within the *hipA* sequence to a position beyond the *speG* orf terminus (Fig. 3).

As for *S. sonnei*, the *in silico* analysis of strain Ss046 indicates that the *speG* locus has been completely lost. The absence of *speG* sequences in all strains from our collection (SsIP1-6, SsZM279 and SsZM328) has been confirmed by Southern blot assays (data not shown). These results are consistent with the well-known clonal nature of *S. sonnei* strains [20].

All together, the observations on the molecular arrangement of the *speG* gene in *Shigella* clearly indicate that *speG* silencing in this microorganism has been attained by convergent evolution. Moreover, our results suggest that this process might have facilitated the adaptation of *Shigella* to the host environment.

### Polyamine pattern in *Shigella* strains

To ascertain whether in *Shigella* spp the loss of a functional *speG* may have altered the polyamine pattern, we analysed the intracellular polyamine level of eight *Shigella* strains by means of HPLC. To this end, *S. flexneri* M90T [31] and SfZM49, *S. sonnei* SsIP3 and SsIP4, *S. dysenteriae* Sd96.29 and Sd4105.65, and *S. boydii* Sb483 and Sb485 were selected as representatives of the diverse arrangements of the *speG* locus (Fig. 3 and Table S1) and grown in polyamine-free medium. Despite the high homology between *Shigella* and *E. coli*, the analysis reveals several relevant differences. In all *Shigella* strains analysed, putrescine and spermidine predominate, while cadaverine, spermidine and acetylspermidine are absent (Table 2). The absence of cadaverine in *Shigella* is well documented [23], [32] and is considered as a major pathoadaptive mutation. The absence of endogenous spermine is not surprising since it has been reported also in *E. coli* [2], which is considered as the commensal ancestor of *Shigella* [20]. As far as spermidine is concerned, interestingly its concentration in *Shigella* was found to be approximately 2- to 3-fold higher than in *E. coli* MG1655. This is almost certainly due to *speG* inactivation and, consequently, to the lack of conversion of spermidine to acetylspermidine. Finally, the polyamine patterns observed in *S. flexneri* SfZM49 and *S. boydii* Sb485 indicate that, despite the presence of specific bands in Southern blot assays (data not shown), *speG* is inactive in these strains too. Based on these data, the absence of acetylspermidine in *Shigella* and the consequent spermidine accumulation can be regarded as a new biochemical feature related to the absence of a functional *speG* gene.

**Table 2:** Analysis of polyamine content in different *Shigella* strains.

	*E. coli*	*S. flexneri*		*S. boydii*		*S. sonnei*		*S. dysenteriae*	
polyamine	MG1655	M90T	SfZM49	Sb483	Sb485	SsIP3	SsIP4	Sd96.29	Sd4105.65
N-SPD	8.2±0.9	n.d.	n.d.	n.d.	n.d.	n.d.	n.d.	n.d.	n.d.
PUT	38.5±2.5	30.8±0.8	21.6±1.2	25.6±1.3	24.8±1.5	17.8±0.9	32.4±1.3	44.5±2.1	25.4±1.0
CAD	7.6±0.7	n.d.	n.d.	n.d.	n.d.	n.d.	n.d.	n.d.	n.d.
SPD	8.6±1.1	18.7±0.5	18.6±0.9	17.4±0.7	22.4±0.9	23.1±0.6	27.5±0.5	22.2±0.8	25.3±0.7

Values reported are in nmol per mg of total proteins and represent the average ± standard deviations (n.d. = not detected). N-SPD: Acetyl spermidine; PUT: Putrescine; CAD: Cadaverine; SPD: spermidine.

### Spermidine accumulation increases resistance to oxidative stress

**Figure 4:**
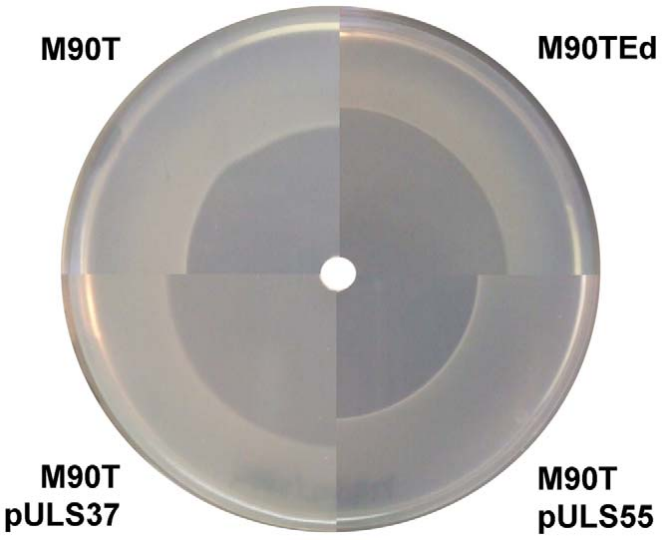
The absence of a functional *speG* gene in *Shigella* increases survival to the oxidative stress. Effect of hydrogen peroxide on *S. flexneri* wild type strain M90T (top left), on its *speE*-defective derivative M90TEd (top right) and on M90T complemented with the entire *ynfB*-*speG* operon (plasmid pULS37, bottom right) or only the *speG* gene (plasmid pULS55, bottom left). A clear difference can be appreciated in the halo of inhibition around the paper disk soaked with 5 µl of H_2_O_2_ 30 w.t. % sol. in water (Sigma-Aldrich); sectors of four agar plates are shown at the same enlargement.

It is known that polyamines play a role in response to oxidative stress [33], [34]. In order to investigate on the effect of spermidine accumulation upon *speG* inactivation, under oxidative stress in *Shigella*, we deleted the *speE* gene, coding for the SpeE protein responsible for spermidine synthetase (Fig. 1), constructing M90TEd, a *Shigella* M90T derivative unable to synthesize spermidine. We then compared M90T, M90TEd and M90T complemented with a plasmid carrying the entire *ynfB*-*speG* operon (pULS37) or the *ynfB* gene alone (pULS55), for survival on minimal medium agar plates in the presence of H_2_O_2_. Plasmids used to this end are derivatives of the low copy plasmid pACYC184 [35], in order to minimize the copy number effects. Measurement of growth halos after 18 hours at 37°C indicated a higher sensitivity to H_2_O_2_ for M90TEd and M90T pULS37 as compared to M90T and M90T pULS55 (Fig. 4). In addition, the oxidative stress resistance was not altered in the M90TEd background by the introduction of pULS37 and pULS55 plasmids (data not shown). This suggests that in M90T spermidine accumulation contributes to increased survival during oxidative stress.

**Figure 5:**
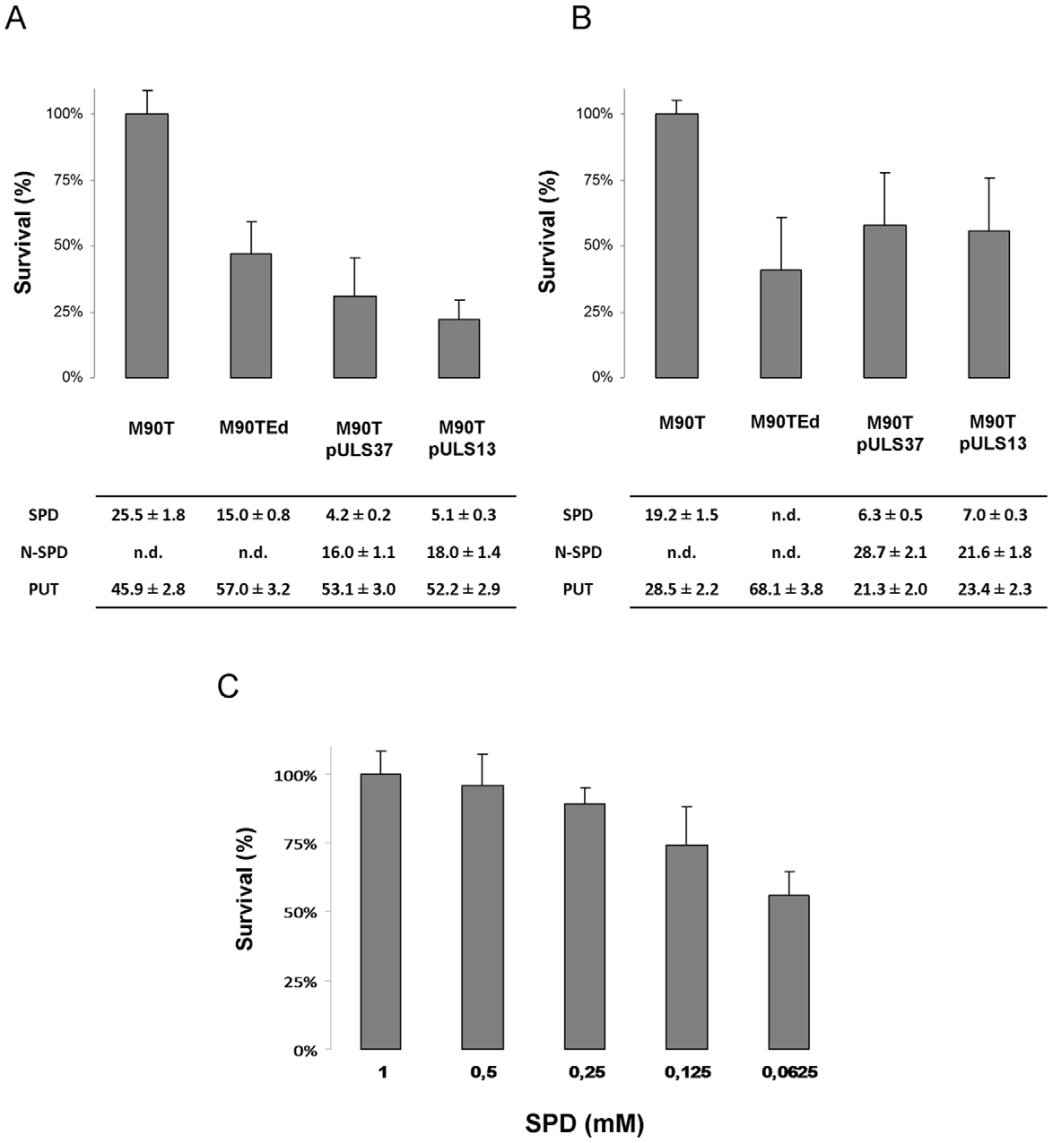
Spermidine involvement in response to oxidative stress in a *S. flexneri* background. *S. flexneri* M90T and its derivatives unable to synthesize spermidine (M90TEd) or carrying either the entire *ynfB-speG* operon (M90T pULS37) or only the promoter proximal *ynfB* gene (M90T pULS13) were grown in LB (panel A) or in M9 minimal medium (panel B) in the presence of H_2_O_2_. Survival is expressed as the percentage relative to the *S. flexneri* M90T wild type strain. The polyamine content of *S. flexneri* strains, obtained by HPLC analysis, is reported in the tables below panels A and B. Values are expressed as nmol/mg of protein. Panel C: *S. flexneri* M90TEd was grown in polyamine-free medium (M9) supplemented with increasing amounts of exogenous spermidine. Survival is expressed as the percentage relative to the M90TEd strain grown in spermidine-free medium (set to 100%). Error bars display the standard deviations relative to at least three independent experiments.

Next, a deeper analysis of the relative survival of M90T, M90TEd and M90T pULS37 under oxidative stress was carried out on strains grown in LB. To confirm that the observed effect was mediated only by *speG* inactivation, we cloned the *speG* coding sequence of MG1655 downstream of a *tac* promoter, obtaining plasmid pULS13, and we also used the M90T pULS13 strain in this analysis. Setting the survival of M90T as 100%, the relative survival of M90T complemented with *speG* (pULS13) or with the *ynfB-speG* operon (pULS37) drops to 23% and 31%, respectively (Fig. 5A top panel). Despite its inability to synthesize spermidine, strain M90TEd exhibited 54% relative survival. Analysis of the polyamine content reveals that the reduced survival of the M90T strains complemented with *speG*-containing plasmids is paralleled by a low level of intracellular spermidine (Fig. 5A bottom panel). The higher level of spermidine in M90TEd is likely to be dependent on its uptake from the LB medium, which we have measured as containing 2.4 µM spermidine, and may be mediated by the conserved spermidine-preferential uptake system consisting of the PotA-D proteins [36]. Therefore, we repeated the experiments in M9, which we verify to be a polyamine-free medium. Under these conditions, we confirmed that the presence of a functional *speG* gene reduces survival to oxidative stress and, in addition, we observed that the strain impaired in spermidine synthesis (M90TEd) displays the lowest survival (Fig. 5B top panel). Hence, in *S. flexneri* a direct correlation exists between cellular spermidine levels and oxidative stress resistance. No correlation was observed for the other polyamines involved. Higher putrescine concentration in M90TEd, both in LB and in M9 media, is not related to the outcome of relative survival to oxidative stress (Fig. 5AB). Moreover, the lack of acetylspermidine in M90T and M90TEd strains does not account for the different relative survival to oxidative stress (Fig. 5A, 5B).

To further and definitively confirm the relationship between intracellular spermidine and oxidative stress resistance in *Shigella*, we analysed the survival of M90TEd grown under oxidative stress in polyamine-free medium after addition of exogenous spermidine. Under this growth condition, the intracellular spermidine level depends exclusively on spermidine uptake. As reported in Fig. 5C, survival decreases with decreasing spermidine concentration, strongly supporting that spermidine and oxidative stress resistance are strictly connected. In *E. coli*, the addition of exogenous spermidine and putrescine (the spermidine precursor) stimulates the expression of the OxyR and *katG* protein, both involved in cellular defence against oxidative stress [37]. OxyR is the global regulator of oxidative stress and acts as positive transcriptional activator, among others, of the *kat*G gene encoding hydroperoxidase I, which catalyses the conversion of H_2_O_2_ to water and oxygen [33]. Starting from this observation, we can hypothesize that the higher concentration of spermidine in the *Shigella* wild type strain, compared to that in the *spe*G-complemented strain, could increase the expression of the *kat*G gene (through OxyR induction) and explain the higher oxidative stress resistance exhibited. In order to verify this hypothesis, we decided to check the *katG* transcriptional activity in M90T and in its *speG*-complemented derivative (M90T pULS13) by means of a real time PCR assay. Interestingly, *katG* mRNA transcription results 8 times higher than that observed in the *speG*-complemented strain. This supports the hypothesis that spermidine accumulation in *Shigella* strains promotes higher expression of the *katG* gene, thereby conferring this microorganism an evolutionary advantage in the response to oxidative stress.

### The patho-adaptative nature of speG defectiveness in *Shigella*

**Figure 6:**
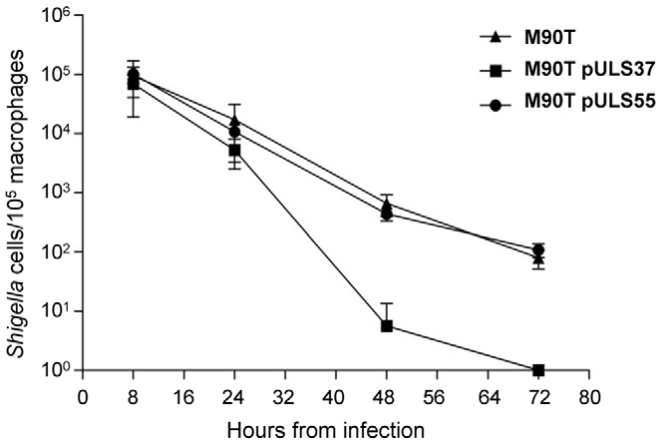
Loss of *speG* confers *Shigella* an increased fitness within murine peritoneal macrophages. Time course of intracellular survival within murine peritoneal macrophages of *S. flexneri* M90T and its derivatives complemented either with the entire *ynfB-speG* operon (plasmid pULS37) or with the *ynfB* gene (plasmid pULS55). The data are the average ± standard deviation of the number of viable intracellular bacteria per 10^5^ macrophages from three independent experiments each in triplicate. ▴, *S. flexneri* M90T; ▪ M90T pULS37; • M90T pULS55.

It is widely accepted that the oxidative stress response may explain the ability of bacterial cells to survive within macrophages [38], [39]. Interestingly, during the first steps of the invasion process *Shigella* is able to persist within macrophages [40]. We used a well-established assay based on infecting BALB/c mice intra-peritoneally, recovering infected peritoneal macrophages and monitoring the survival of intracellular bacteria within *in vitro*-maintained macrophages over a 72 h period [39], [41]. We compared the intracellular survival of *S. flexneri* M90T with that of isogenic strains containing plasmids carrying the entire *ynfB-speG* operon (pULS37) or only *ynfB* (pULS55). No significant difference was observed among strains recovered 8 h after infection, suggesting that all strains are equally able to infect macrophages (Fig. 6). This reinforces previous experimental observations, obtained by plaque assays (data not shown), that indicated no difference in infectivity and spreading among *S. flexneri* M90T, M90T pULS37 and M90T pULS55 on HeLa cell monolayers. The ability to survive intracellularly 24 h, 48 h, and 72 h after infection, of M90T and M90T pULS55 decreased to a comparable extent, whereas strain M90T pULS37 is significantly more susceptible to macrophage killing from the 24 h time point on (Fig. 6). This indicates that in *Shigella* restoration of SpeG activity reduces the ability of bacterial cells to withstand hostile conditions within macrophages.

Further evidence supporting this conclusion was obtained by means of an *in vitro* competitive assay analysing the survival within macrophages of *S. flexneri* M90T complemented with the entire *ynfB-speG* operon (pULS37) or only with the *ynfB* gene (pULS55). Strain M90T and its derivatives carrying pULS37 or pULS55 were grown to OD_600_ 0.3–0.4, mixed and used to infect a murine macrophage cell line (J774). Bacterial survival was monitored two hours after infection by lysing the macrophages and plating appropriate dilutions on LB plates. To discriminate M90T from its pULS-derivatives, 200 colonies were replicated on LB plates containing tetracycline. As shown in Table 3, when comparing M90T with M90T pULS55 the competitive index (C.I.) corresponds to 1.11 and 1.05 at 1 h and 2 h, respectively, while it drops to 0.74 (1 h) or 0.43 (2 h) when comparing M90T with M90T pULS37 indicating that the M90T strain, in the absence of a functional *speG* gene, is more competitive for survival in macrophages. All together, these observations indicate that the evolutionary acquired absence of SpeG activity in *Shigella* confers the bacterium with an increased capability to defy antagonistic host environments. Thus, it can be assumed that the major functional impact of the lack of *speG* resides in its pathoadaptive significance.

**Table 3:** Competitive infection assay in macrophages.

	Competitive Index (C.I.)	
Strain vs M90T_(wt)_	1 h	2 h
M90T pULS55_(*ynfB*)_	1.11±0.22	1.05±0.30
M90T pULS37_(*ynfBspeG*)_	0.73±0.18	0.44±0.12

## Discussion

The evolution of bacterial pathogens from harmless ancestors mainly depends on the acquisition of virulence gene clusters on plasmids, phages and pathogenicity islands by lateral gene transfer [42], [43]. Complementary to this process is the progressive adaptation to a specific niche by pathoadaptive events involving mutations, rearrangements or deletions of genes unnecessary, or even deleterious, for optimal fitness to the new environment [23]. These events usually involve the concomitant arrival or loss of regulatory factors and this might modify the transcriptional profile of the host to a significant extent. In this work, we have analysed the genetic consequences of the uptake of the *virF* gene by the harmless ancestor of *Shigella*, the gut commensal *E. coli*. A crucial step in the evolution of *Shigella* from *E. coli* is the acquisition of the large pINV plasmid, which carries the genes required for the invasion of the colonic epithelium [20]. The primary regulator of these genes, the *virF* gene, encodes an AraC-like transcriptional activator that resides on pINV. The results we report in the present study provide evidence that the activity of VirF is not restricted to the regulation of the virulence system: many other chromosomal *E. coli* genes are subject to direct or indirect activation by *virF*.

Comparative sequence analysis of the genomes of *Shigella* strains, available in current databases, reveals that VirF-activated genes fall into two different groups: one containing genes still conserved in *Shigella* and the other containing genes which are inactivated or deleted in *Shigella*. In the first group, among the genes more susceptible to *virF* induction, we found the genes encoding the heat shock proteins IbpA, GroESL, HtpG, DnaK and Lon. Interestingly, HtpG, which belongs to the HSP90 family, is implicated in the inflammatory response of *Shigella* in infected mice and is considered a potential subunit vaccine candidate against shigellosis [44]. These observations suggest that VirF, besides operating as a primary virulence regulator, also activates genes whose products contribute to better withstanding of adverse conditions inside the host.

The existence of a group of genes activated by VirF in *E. coli*, but no longer present or non-functional in *Shigella*, is an intriguing result. We focused our attention on the *speG* gene, the best characterized one, whose product is involved in the biosynthesis of polyamines. Putrescine, cadaverine, spermine and spermidine are implicated in several aspects of cellular life. They affect membrane permeability, gene expression, intracellular signalling, oxidative stress resistance, pH stress resistance and apoptosis through non-covalent interactions with nucleic acids or specific interplay with proteins [45]. The SpeG protein is a spermidine-acetyltransferase (SAT), which transfers an acetyl group to either the N-1 or the N-8 position of spermidine. Acetylation converts polyamines, in particular spermidine, into a physiologically inert form. Acetylpolyamines cannot substitute for polyamine in RNA binding, in growth enhancement of *E. coli* polyamine-deficient mutants nor in the stimulation of *in vitro* translation [46]. Acetylation has a major impact on spermidine catabolism: the lack of SAT activity produces spermidine accumulation in *E. coli* [8].

In order to evaluate the extent of *speG* inactivation in *Shigella*, we integrated the genomic observations with a specific comparative study of the *speG* locus of several *Shigella* strains (Table S1). The *speG* gene is inactivated in all strains analysed. Inactivating mutations include point mutations and entire gene deletions, suggesting the existence of a strong selective pressure towards the loss of SpeG function in *Shigella*. The absence of *speG* hybridization signals in all *S. sonnei* strains analysed and in some strains of *S. flexneri* (SfZM53) and *S. boydii* (Sb51 and Sb485) may represent the final results of several progressive steps. Between the two extremes, i.e. the presence of a complete *ynfB-speG* operon with a missense mutation and the deletion of the entire *speG* locus, we observe a series of genetic rearrangements, mainly induced by *IS* elements. In particular, the results obtained by the analysis of *S. boydii* nicely exemplify how a progressive erosion of the *speG* locus has occurred after accumulation of *IS* sequences next to the *speG* gene (Fig. 3), leaving a non-functional truncated 3′ sequence. The sequence of the *speG* locus in the *S. boydii* strains analysed in this work reveals at least three subsequent steps, from an initial *IS*911 insertion in the *speG* sequence, strains Sb481 and Sb483, to the complete deletion of the *speG* locus, strains Sb484 and Sb51.

Since the molecular rearrangements we observed clearly hint at the existence of selective pressure towards the loss of *speG* functionality, we looked at the consequences of these mutations on the intracellular polyamine balance. The results obtained by considering a pool of eight representative *Shigella* strains (Table 2) show that a common feature is the absence of spermine, cadaverine and acetylated spermidine, coupled to the presence of putrescine and spermidine. In particular, the endogenous spermidine concentration is 2- to 3-fold higher in *Shigella* strains as compared to *E. coli* K12. While the lack of spermine in *E. coli* is well known [2] and the deficiency of cadaverine in *Shigella* is documented [32], the absence of the acetylated form of spermidine, determined by *speG* defectiveness, is a novel finding. The lack of SAT activity, the presence of efficient systems for spermidine and putrescine uptake [36] and the likely absence of known efficient spermidine secretion systems cause accumulation of this polyamine. Moreover, neither a spermidine deacetylating activity nor a spermidine or polyamine oxidase activity can be detected in *E. coli* or in *Shigella*. As for the presence of putrescine, spermidine accumulation is known to inhibit ornithine decarboxylase and arginine decarboxylase, both involved in putrescine biosynthesis, maintaining the endogenous putrescine concentration at physiological levels [47].

Besides constituting a new physiological trait of *Shigella*, how does the abundance of spermidine in this microorganism relate to its virulence specificity? In this context, it is worth stressing that other polyamines are involved in *Shigella* virulence. In particular, the addition of exogenous putrescine, as well as of methionine and arginine (both implicated in putrescine/spermidine biosynthesis) can restore virulence in *S. flexneri* mutants that are unable to synthesize modified nucleosides required for tRNA synthesis [48]. More recently, putrescine has been shown to relieve the ornithine repression exerted on *Shigella* virulence in minimal medium [49]. Overall, during the last years an increasing number of studies related to polyamines in bacteria report new insights about the active role of polyamines during diverse steps of the pathogenic process of different virulent species [50]. Therefore, we asked whether spermidine accumulation, due to *speG* inactivation, is advantageous for cellular physiology, for the full expression of virulence determinants and for the correct progress of the virulence program. First, we tested the oxidative stress resistance in *Shigella* since this microrganism experiences a certain degree of oxidative stress within the macrophage cytosol [51] and the role of polyamines in this process, although not yet completely characterized, is well documented [34]. Polyamines are involved in *katG* expression since they favor the translation of OxyR, a key regulator of the stress response [33]. By treating wild type *speE*-defective and *speG*-complemented *S. flexneri* strains with H_2_O_2_, we observed a direct correlation between intracellular spermidine concentration and oxidative stress resistance (Fig. 5A, B, C). In this context, we observed that the absence of a functional SpeG in *Shigella* induces an increase of KatG expression. Moreover, it is worth remembering that cadaverine is reported as the best polyamine providing bacterial resistance to oxidative stress [34], that *Shigella* is typically a cadaverine-defective microorganism [22], [23] and that, while cadaverine is secreted, spermidine is preferentially retained intracellularly [36]. Based on these observations and on the possible functional complementarity among different polyamines in cell [2], the increase of spermidine in *Shigella* may compensate for the absence of cadaverine in order to maintain an effective response to oxidative stress.

As for the impact of spermidine accumulation on the expression of virulence determinants and on the progress of the virulence program, our plaque assay experiments on HeLa cells (data not shown) reveal no difference in infectivity and spreading between *S. flexneri* and its *speG*-complemented derivative. On the other hand, the outcome of intracellular macrophage survival assays, performed in mouse peritoneal macrophages (Fig. 6), and of a competitive-infection assay on J774 macrophage cell culture (Table 3), shows a decrease of survival properties in the *speG*-complemented *Shigella* strains. These data suggest that bacterial survival inside macrophages may also depend on the ability of *Shigella* to exploit the polyamine-mediated neutralization of the oxidative stress to which these bacteria are exposed into the macrophage upon infection.

All together our observations reveal the patho-adaptative nature of *speG* inactivation in *Shigella*, suggesting a supportive role of this adaptation in the pathogenicity of *Shigella*. It is tempting to speculate that, during the evolutionary transition from *E. coli* to *Shigella*, the acquisition of *virF* by means of lateral gene transfer might have caused an increased expression of *speG*, thus lowering the intracellular spermidine content. This new set up could have hindered the survival strategies of the bacterium within the infected host cells. Hence, *speG* inactivation would have been favoured in order to increase the intracellular levels of spermidine. This is supported by the presence of an efficient putrescine/spermidine importer [36] and by the absence of an effective spermidine secretion system in *Shigella*. It is worth stressing that the recently identified MdtIJ protein complex, belonging to the small multidrug resistance (SMR) family and encoded by the *mdtIJ* operon, is able to secrete spermidine effectively only when cloned on a multicopy plasmid [52].

*Shigella* is acquired by oral contamination and is able to cross different parts of the digestive tract. In the intestinal lumen, the major polyamines (putrescine and cadaverine) are produced mainly by bacteria and are mostly free. The existence of a negative putrescine gradient from the jejunum to the ileum has been surmised due to the rapid absorption of luminal polyamines by the intestinal mucosa [53]. On account of these observations, we hypothesize that *speG* inactivation enables *Shigella* to compete with the intestinal mucosa for putrescine uptake so, when *Shigella* reaches the polyamine-poor colon and crosses the epithelial mucosa, it survives inside resident macrophages, even by virtue of the high level of intracellular spermidine synthesized from by the absorbed putrescine. This step is then followed by the induction of macrophage apoptosis and by the invasion of enterocytes, the final target of this pathogen. Other studies are in progress to investigate the involvement of cytoplasmatic spermidine accumulation in different steps of the S*higella*'s virulence program, taking account of the involvement of this polyamine in gene regulation and of its pro-apoptotic and immuno-modulating properties [54], [55].

## Materials and Methods

### Bacterial Strains, plasmids and general procedures

The bacterial strains used are listed in Table S1. *E. coli* strains ULS153 and ULS117 and *S. flexneri* strains 2457TFd and M90TEd were obtained using the one-step method of gene inactivation [56]. Strain ULS153, carrying a deletion of the *lacZ* gene, and strain ULS117, carrying a deletion of the entire *speG* gene, were constructed by transforming MG1655 pKD46 with amplicons obtained using plasmid pKD13 as template and the oligo pairs *dlf/dlr* or *dgf*/*dgr*. The same procedure was used to construct the *speE* defective *S. flexneri* strain M90TEd (oligo pair *def*/*der*) and the *virF* defective *S. flexneri* strain 2457Fd (oligo pair *dff*/*dfr*).

Bacteria were grown in Luria broth (LB), Brain Infusion Heart (BHI) or M9 minimal medium [57]. When required, 0.125 to 1 mM spermidine and 10 µg/ml nicotinic acid were included in M9 medium. Antibiotics were used at the following concentrations: ampicillin, 100 µg/ml; chloramphenicol, 30 µg/ml; kanamycin, 30 µg/ml; tetracycline, 5 µg/ml. Solid media contained 1.6% agar.

β-galactosidase assays were performed as previously described [57] on sodium dodecyl sulfate-chloroform-permeabilized cells grown in LB supplemented with ampicillin. Units of β-galactosidase were calculated by the method of Miller [57]. PCR reactions were routinely performed using Dreamtaq DNA polymerase (Fermentas). Ex taq DNA polymerase (Takara) was adopted to obtain longer transcripts and high fidelity. Oligos used are listed in Table S3. Genomic DNA purifications were performed using the “mi-Bacterial Genomic DNA isolation kit” (MetaBion). DNA sequencing was performed by Synergene Biotech.

### Plasmid construction

Plasmid pULS7 was constructed by cloning a fragment carrying the *ynfB-speG* promoter region and the first 35 codons of the MG1655 *speG* gene into plasmid pRS414, which is a vector suitable for generating protein fusions: the first eight codons of the *lacZ* gene were removed and a multicloning site has been inserted upstream the *lacZ* gene [58]. The amplicon obtained with oligo pair *pgf*/*pgr*, modified to contain a *BamHI* site, and MG1655 DNA as template was digested with *BamHI* and cloned into *BamHI* linearized pRS414.

Plasmids pULS11 and pULS12, containing the entire *ynfB-speG* operon, were constructed by cloning into pGEM-T easy a DNA fragment obtained by PCR with the oligo pair *pgf*/*ygt* and total DNA of MG1655 or *S. dysenteriae* Sd12 as template.

In order to construct plasmid pULS37, we subcloned the *EcoRI* fragment containing the *ynfB-speG* region from pULS11 to the pACYC184 *EcoRI* site. pULS55 was obtained by cloning into pGEM-T easy a functional copy of the *ynfB* gene with its regulatory region, obtained by PCR with the oligo pair *pgf/pgr* and MG1655 DNA as template. The *EcoRI* fragment containing the *ynfB* gene was then subcloned from pGEM-T easy to the pACYC184 *EcoRI* site. Plasmids pMY6520R and pMY6504R were obtained by re-ligating a *HindIII* partial digest of pMYSH6520 and pMYSH6504 plasmids in order to delete the *virF* gene. The loss of *virF HindIII* fragments was verified by sequencing.

To monitor *speG* expression independently from *ynfB* transcription, we cloned the *speG* gene into pGIP7, a pACYC184 vector carrying a *tac* promoter and the LacI encoding gene [59]. To this end, a PCR fragment obtained using oligo pair *gof*/*gor* and MG1655 DNA as template was digested with *BamHI* and cloned into the pGIP7 *BamHI* site, thus obtaining plasmid pULS13.

### Polyamine quantification

Polyamines were extracted from suspensions of bacteria with 0.25 M percloric acid containing 5 µM 1,6-diaminehexane as a polyamine internal standard. They were then quantified after derivatization with dansyl cloride and separation by HPLC. The simultaneous fluorimetric determination of intracellular polyamines was performed by reverse-phase high-performance liquid chromatography [60]. Polyamine concentration in the total cellular homogenates was normalized with respect to the corresponding protein content and expressed as nmol/mg of proteins.

### Microarray analysis

Bacterial strains MG1655 pMY6504R and MG1655 pMYSH6504 were grown at 37°C in 10 ml LB to OD_600_∼0.6. Cells were immediately chilled and total cellular RNA was isolated by hot phenol extraction [61] and DNase I-treatment for 30 min at 37°C. RNA pellets were suspended in 30 µl diethylpyrocarbonate (DEPC)-treated water. Cy5-dCTP and Cy3-dCTP (GE Healthcare) were used to synthetize labelled cDNA using the direct labelling procedure of the LabelStar™ Array Kit (Qiagen). The *E. coli* K12-V2 Array (MWG), hybridized to labelled probe pools, were scanned and quantified using a ScanArray lite scanner (Packard Bioscience) and the ScanArray Express software. Two arrays were used and analysed as replicates. Resulting data were processed by Global Lowess normalization and averaged using J-Express software (MolMine AS). We filtered the data to exclude artefacts and low signal spots. Finally, only genes with an error rate lower than 30% and with ratio of 2 and above were considered. Normalized ratio data of microarray experiment is provided in Table S2. All microarray data reported in the manuscript is described in accordance with MIAME guidelines and the data from the experiments are deposited in GEO (accession no. GSE30207).

### Real Time PCR

Total RNA was extracted as previously described and cDNA synthesis was performed using the High Capacity cDNA Reverse Transcription Kit from Applied Biosystems in a 20 µl reaction mix containing 20 µg total RNA. Real time quantitative PCR was performed with the aid of a 7300 Real-Time PCR System (Applied Biosystems) in a 30 µl reaction mix containing 2 µl cDNA and Power SYBR®Green PCR Master Mix (Applied Biosystems). At least three wells were run for each sample. The relative amounts of *speG* transcript was analysed using the 2^−ΔΔCt^ method [62] and the results were indicated as a n-fold increase relative to the reference sample. Primers for the *mdh* transcript, used as endogenous control, and for *speG* and *katG* transcripts were designed with the aid of the Primer Express® software v2.0 (Applied Biosystems) and experimentally validated for suitability to the 2^−ΔΔCt^ method. The following oligos pairs were used: *mdf*/*mdr* for the *mdh* gene; *rgf/rgr* for the *speG* gene and *kgf/kgr* for *katG* gene.

### Bacterial susceptibility to oxidative stress

Bacterial susceptibility to oxidative stress was tested as follow: bacterial cultures were grown overnight, diluted in fresh LB or M9 minimal medium and allowed to growth to OD_600_ 0.6–0.8.

15 ml for each culture were centrifuged and pellets suspended in 1 ml 1× PBS. 1 ml of 1× PBS containing 10 mM H_2_O_2_ was added and left to react for 30 minutes at 37°C. The reaction was stopped by adding Catalase to 0.1 mg/ml (Sigma-Aldrich). The number of bacteria surviving the oxidative stress was then quantified by plating aliquots on LB Agar.

### Survival assays in mouse peritoneal macrophages

Survival of *S flexneri* M90T strain in mouse peritoneal macrophages was tested using an *in vivo–in vitro* infection model as described previously [39]. Briefly, strains M90T and its derivatives M90T pULS37 or pULS55 (Table S1) were grown at 37°C in BHI to OD600∼0.4. *E. coli* strain DH5α grown in LB at 37°C was used as control. The bacteria were harvested by centrifugation and suspended in PBS at 5×10^7^ cells/ml. Male BALB/c mice (10 weeks old) were infected by intra-peritoneal injection of each strain. After a 6 h infection period, peritoneal macrophages were collected by peritoneal lavage, centrifuged and suspended in Dulbecco's modified Eagle's medium (DMEM) containing 10 mM HEPES, 2 mM glutamine, 10% bovine fetal serum, 16 non-essential amino acids, and 150 µg/ml gentamicin. The cell suspension was dispensed into 24-well tissue-culture plates, incubated at 37°C under 5% CO_2_ for 2 h, and bacterial survival was monitored at 24, 48 and 72 h. The animal experiments were performed under a protocol approved by the Institutional Animal Use and Care Committee at Università Cattolica del S. Cuore, Rome, Italy (Permit number: H21, 07/24/2008) and authorized by the Italian Ministry of Health, according to Legislative Decree 116/92, which implemented the European Directive 86/609/EEC on laboratory animal protection in Italy. Animal welfare was routinely checked by veterinarians of the Service for Animal Welfare.

### Culture of macrophages and bacterial infection

The murine macrophage-like cells J774 (American Type Culture Collection, Manassas, VA) were grown in RPMI 1640 (Gibco) medium containing 10% heat-inactivated fetal bovine serum (Euroclone) and 2 mM L-glutamine at 37°C in a humidified 5% CO_2_ atmosphere. For bacterial infection, the cells were seeded in 24-well tissue culture plates (Falcon) at a density of 10^5^ cells/cm^2^ and grown over night at 37°C in fresh medium without antibiotics. Bacterial uptake, survival and replication were measured by a gentamicin protection assay [63]. Before infection, cell monolayers were washed twice with phosphate-buffered saline (PBS; pH 7,2), and the medium was replaced by 1 ml of RPMI 1640 supplemented with 10% heat-inactivated foetal bovine serum. In order to produce a competitive infection, M90T and M90T pULS37 (or M90T and M90T pULS55) were used to simultaneously infect J774 monolayers with at a multiplicity of infection of 100 bacteria per macrophage. After 5 min of centrifugation at 900 rpm and a 15 min incubation at 37°C with 5% CO_2_, the infected macrophages were washed twice with PBS. Fresh cell culture medium containing 25 µg/ml of gentamicin was added to kill extracellular bacteria and the cells were incubated further at 37°C for 1 h and 2 h. To determine the number of intracellular bacteria, the cells were washed once with PBS and lysed by adding 0.5 ml of 1% Triton X-100 (Sigma) to each well for 5 min. Samples were mixed, diluted and plated onto LB agar plates to determine the number of CFU recovered from the lysate. The number of intracellular bacteria was determined after 1 and 2 h of gentamicin treatment and compared to bacteria plated at time zero. To calculate the competitive index (C.I.), the ratios of strains M90T pULS55/M90T and of strains M90T pULS37/M90T recovered from the infected cultures were determined and then normalized by dividing by the corresponding ratio in the initial inoculum.

### Nucleotide sequence accession number

DNA sequence data were compared to known nucleotide and protein sequences using the BLAST server (National Center of Biotechnology Information, Bethesda, Md.). All new sequences of *ynfB speG* regions of *Shigella* strains have been deposited at GeneBank under the following accession number: JF737027, JF737028, JF737029 and JF737030 referred to *S. flexneri* strain M90T, SfZM50, SfZM53 and YSH6000; JF737021, JF737025, JF737026, JF742750 and JF742751 referred to *S. dysenteriae* strain Sd12, Sd16.81, Sd4105.65, SdZM603 and Sd96.29; JF737022, JF737024, JF737020 and JF737023 referred to *S. boydii* strain Sb481, Sb483, Sb411 and Sb484.

## Supporting Information

Table S1 **Bacterial strains and plasmids.**
(DOC)

Table S2 ***E. coli*** **genes induced by** ***Shigella virF*** **gene.**
(DOC)

Table S3 **Oligos used in this study.**
(DOC)

## References

[1] Cohen SS (1997). A guide to the polyamines.

[2] Tabor CW, Tabor H (1985). Polyamines in microorganisms.. Microbiol Rev.

[3] Seiler N (1987). Functions of polyamine acetylation.. Can J Physiol Pharmacol.

[4] Kashiwagi K, Igarashi K (1988). Adjustment of polyamine contents in *Escherichia coli*.. J Bacteriol.

[5] Meng SY, Bennett GN (1992). Nucleotide sequence of the *Escherichia coli cad* operon: a system for neutralization of low extracellular pH.. J Bacteriol.

[6] Kikuchi Y, Kojima H, Tanaka T, Takatsuka Y, Kamio Y (1997). Characterization of a second lysine decarboxylase isolated from *Escherichia coli*.. J Bacteriol.

[7] Xie QW, Tabor CW, Tabor H (1989). Spermidine biosynthesis in *Escherichia coli* promoter and termination regions of the *speED* operon.. J Bacteriol.

[8] Fukuchi J, Kashiwagi K, Yamagishi M, Ishihama A, Igarashi K (1995). Decrease in cell viability due to the accumulation of spermidine in spermidine acetyltransferase-deficient mutant of *Escherichia coli*.. J Biol Chem.

[9] Zhou L, Wang J, Zhang LH (2007). Modulation of bacterial Type III secretion system by a spermidine transporter dependent signaling pathway.. PLoS One.

[10] Shah P, Nanduri B, Swiatlo E, Ma Y, Pendarvis K (2011). Polyamine biosynthesis and transport mechanisms are crucial for fitness and pathogenesis of *Streptococcus pneumoniae*.. Microbiology.

[11] Patel CN, Wortham BW, Lines JL, Fetherston JD, Perry RD (2006). Polyamines are essential for the formation of plague biofilm.. J Bacteriol.

[12] McGinnis MW, Parker ZM, Walter NE, Rutkovsky AC, Cartaya-Marin C (2009). Spermidine regulates *Vibrio cholerae* biofilm formation via transport and signalling pathways.. FEMS Microbiol Lett.

[13] Sturgill G, Rather PN (2004). Evidence that putrescine acts as an extracellular signal required for swarming in *Proteus mirabilis*.. Mol Microbiol.

[14] Allison C, Coleman N, Jones PL, Hughes C (1992). Ability of *Proteus mirabilis* to invade human urothelial cells is coupled to motility and swarming differentiation.. Infect Immun.

[15] Lasbury ME, Merali S, Durant PJ, Tschang D, Ray CA (2007). Polyamine-mediated apoptosis of alveolar macrophages during *Pneumocystis pneumonia*.. J Biol Chem.

[16] Kotloff KL, Winickoff JP, Ivanoff B, Clemens JD, Swerdlow DL (1999). Global burden of *Shigella* infections: implication for vaccine development and implementation of control strategies, Bull.. World Health Organ.

[17] Ashida H, Ogawa M, Mimuro H, Sasakawa C (2009). *Shigella* infection of intestinal epithelium and circumvention of the host innate defense system.. Curr Top Microbiol Immunol.

[18] Sansonetti PJ (2006). The bacterial weaponry: lessons from *Shigella*.. Ann N Y Acad Sci.

[19] Buchrieser C, Glaser P, Rusniok C, Nedjari H, D'Hauteville H (2000). The virulence plasmid pWR100 and the repertoire of proteins secreted by the type III secretion apparatus of *Shigella flexneri*.. Mol Microbiol.

[20] Lan R, Reeves PR (2002). *Escherichia coli* in disguise: molecular origins of *Shigella*.. Microbes Infect.

[21] Sokurenko EV, Hasty DL, Dykhuizen DE (1999). Pathoadaptive mutations: gene loss and variation in bacterial pathogens.. Trends Microbiol.

[22] Prosseda G, Carmela Latella M, Barbagallo M, Nicoletti M (2007). The two-faced role of *cad* genes in the virulence of pathogenic *Escherichia coli*.. Res Microbiol.

[23] Maurelli AT (2007). Black holes, antivirulence genes, and gene inactivation in the evolution of bacterial pathogens.. FEMS Microbiol Lett.

[24] McCormick BA, Fernandez MI, Siber AM, Maurelli AT (1999). Inhibition of *Shigella flexneri*-induced transepithelial migration of polymorphonuclear leucocytes by cadaverine.. Cell Microbiol.

[25] Fernandez MI, Silva M, Schuch R, Walker WA, Siber AM (2001). Cadaverine prevents the escape of *Shigella flexneri* from phagolysosome: a connection between bacterial dissemination ad neutrophil transepithelial signalling.. J Infect Dis.

[26] Prosseda G, Falconi M, Giangrossi M, Gualerzi CO, Micheli G (2004). The *virF* promoter in *Shigella*: more than just a curved DNA stretch.. Mol Microbiol.

[27] Prosseda G, Falconi M, Nicoletti M, Casalino M, Micheli G (2002). Histone-like proteins and the *Shigella* invasivity regulon.. Res Microbiol.

[28] Blattner FR, Plunket G, Bloch CA, Perna T, Burland V (1997). The complete sequence of *Escherichia coli* K-12.. Science.

[29] Sakai T, Sasakawa C, Makino S, Yoshikawa M (1986). DNA sequence and product analysis of the *virF* locus responsible for congo red binding and cell invasion in *Shigella flexneri* 2a.. Infect Immun.

[30] Casalino M, Nicoletti M, Salvia A, Colonna B, Pazzani C (1994). Characterization of endemic *Shigella flexneri* strains in Somalia: antimicrobial resistance, plasmid profiles, and serotype correlation.. J Clin Microbiol.

[31] Vaudaux P, Waldvogel FA (1979). Gentamicin antibacterial activity in the presence of human polymorphonuclear leukocytes.. Antimicrob Agents Chemother.

[32] Day WA, Fernández RE, Maurelli AT (2001). Pathoadaptive mutations that enhance virulence: genetic organization of the *cadA* regions of *Shigella spp*.. Infect Immun.

[33] Jung IL, Kim IG (2003). Transcription of *ahpC*, *katG*, and *katE* genes in *Escherichia coli* is regulated by polyamines: polyamine-deficient mutant sensitive to H_2_O_2_-induced oxidative damage.. Biochem Biophys Res Commun.

[34] Chattopadhyay MK, Tabor CW, Tabor H (2003). Polyamines protect *Escherichia coli* cells from the toxic effect of oxygen.. Proc Natl Acad Sci U S A.

[35] Sambrock J, Russel DW (2001). Molecular cloning: a laboratory manual, 3^rd^ ed.

[36] Igarashi K, Kashiwagi K (2009). Polyamine transport in bacteria and yeast.. Biochem J.

[37] Tkachenko AG, Nesterova LY (2003). Polyamines as modulators of gene expression under oxidative stress in *Escherichia coli*.. Biochemistry (Mosc).

[38] Storz G, Imlay JA (1999). Oxidative stress.. Curr Opin Microbiol.

[39] Verneuil N, Sanguinetti M, Le Breton Y, Posteraro B, Fadda G, Auffray Y (2004). Effects of the *Enterococcus faecalis hypR* gene encoding a new transcriptional regulator on oxidative stress response and intracellular survival within macrophages.. Infect Immun.

[40] Ogawa M, Sasakawa C (2006). Intracellular survival of *Shigella*.. Cell Microbiol.

[41] Gentry-Weeks CR, Karkhoff-Schweizer R, Pikis A, Estay M, Keith JM (1999). Survival of *Enterococcus faecalis* in mouse peritoneal macrophages.. Infect Immun.

[42] Ochman H, Lawrence JG, Groisman EA (2000). Lateral gene transfer and the nature of bacterial innovation.. Nature.

[43] Dobrindt U, Hacker J (2001). Whole genome plasticity in pathogenic bacteria.. Curr Opin Microbiol.

[44] Bu X, Zhu L, Liu X, Zhao G, Feng E (2008). HtpG protein of *Shigella flexneri* 2a strain 2457T evokes inflammatory response in mice.. Wei Sheng Wu Xue Bao.

[45] Wortham BW, Patel CN, Oliveira MA (2007). Polyamines in bacteria: pleiotropic effects yet specific mechanisms.. Adv Exp Med Biol.

[46] Kakegawa T, Guo Y, Chiba Y, Miyazaki T, Nakamura M (1991). Effect of acetylpolyamines on in vitro protein synthesis and on the growth of a polyamine-requiring mutant of *Escherichia coli*.. J Biochem.

[47] Bachrach U, Heimer YM (1989). The Physiology of Polyamines.

[48] Durand JM, Björk GR (2003). Putrescine or a combination of methionine and arginine restores virulence gene expression in a tRNA modification-deficient mutant of *Shigella flexneri*: a possible role in adaptation of virulence.. Mol Microbiol.

[49] Durand JM, Björk GR (2009). Metabolic control through ornithine and uracil of epithelial cell invasion by *Shigella flexneri*.. Microbiology.

[50] Shah P, Swiatlo E (2008). A multifaceted role for polyamines in bacterial pathogens.. Mol Microbiol.

[51] Lucchini S, Liu H, Jin Q, Hinton JCD, Yu J (2005). Transcriptional adaptation of *Shigella flexneri* during infection of macrophages and epithelial cells: insight into the strategies of a cytosolic bacterial pathogen.. Infect Immun.

[52] Higashi K, Ishigure H, Demizu R, Uemura T, Nishino K (2008). Identification of a spermidine excretion protein complex (MdtJI) in *Escherichia coli*.. J Bacteriol.

[53] Osborne DL, Seidel ER (1990). Gastrointestinal luminal polyamines: cellular accumulation and enterohepatic circulation.. Am J Physiol.

[54] Pérez-Cano FJ, Franch A, Castellote C, Castell M (2003). Immunomodulatory action of spermine and spermidine on NR8383 macrophage line in various culture conditions.. Cell Immunol.

[55] Mariggiò MA, Vinella A, Pasquetto N, Curci E, Cassano A (2004). In vitro effects of polyamines on polymorphonuclear cell apoptosis and implications in the pathogenesis of periodontal disease.. Immunopharmacol Immunotoxicol.

[56] Datsenko KA, Wanner BL (2000). One-step inactivation of chromosomal genes in *Escherichia coli* K-12 using PCR products.. Proc Natl Acad Sci U S A.

[57] Miller JH (1992). A short course in bacterial genetics.

[58] Simons RW, Houman F, Kleckner N (1987). Improved single and multicopy *lac*-based cloning vectors for protein and operon fusions.. Gene.

[59] Falconi M, Prosseda G, Giangrossi M, Beghetto E, Colonna B (2001). Involvement of FIS in the H-NS-mediated regulation of *virF* gene of *Shigella* and enteroinvasive *Escherichia coli*.. Mol Microbiol.

[60] Matés JM, Márquez J, García-Caballero M, Núñez de Castro I (1992). Simultaneous fluorometric determination of intracellular polyamines separated by reversed-phase high-performance liquid chromatography.. Agents Actions.

[61] von Gabain A, Belasco JG, Schottel JL, Chang AC, Cohen SN (1983). Decay of mRNA in *Escherichia coli*: investigation of the fate of specific segments of transcripts.. Proc Natl Acad Sci U S A.

[62] Livak KJ, Schmittgen TD (2001). Analysis of relative gene expression data using real-time quantitative PCR and the 2(−Delta Delta C(T)) Method.. Methods.

[63] Sansonetti PJ, Kopecko DJ, Formal SB (1982). Involvement of a plasmid in the invasive ability of *Shigella flexneri*.. Infect Immun.

