# Correction: A New Piece of the *Shigella* Pathogenicity Puzzle: Spermidine Accumulationby Silencing of the *speG* Gene

**DOI:** 10.1371/annotation/272940da-34b6-4cd7-8794-ecb5df8d7cc2

**Published:** 2012-05-31

**Authors:** Marialuisa Barbagallo, Maria Letizia Di Martino, Lucia Marcocci, Paola Pietrangeli, Elena De Carolis, Mariassunta Casalino, Bianca Colonna, Gianni Prosseda

There was a typographical error in the title. The correct title is: A New Piece of the *Shigella* Pathogenicity Puzzle: Spermidine Accumulation by Silencing of the *speG* Gene. The correct citation is: Barbagallo M, Di Martino ML, Marcocci L, Pietrangeli P, De Carolis E, et al. (2011) A New Piece of the *Shigella* Pathogenicity Puzzle: Spermidine Accumulation by Silencing of the *speG* Gene. PLoS ONE 6(11): e27226. 

