# Supplementary material for: A New Piece of the Shigella Pathogenicity Puzzle: Spermidine Accumulationby Silencing of the speG Gene
Source: PLoS One. 2011 Nov 10;6(11):e27226. doi: 10.1371/journal.pone.0027226 (PMC3213128; doi:10.1371/journal.pone.0027226)
Supplement: Table S1 — Bacterial strains and plasmids. (DOC) [file pone.0027226.s001.doc]

Table S1. Bacterial strains and plasmids

| Strain or plasmids | Relevant features | Source |
| --- | --- | --- |
| Strains |  |  |
| DH5α | *F^-^, φ80dlacZΔM15, Δ(lacZYA-argF)U169, deoR, recA1, endA1, hsdR17(rk^-^, mk^+^), phoA, supE44, λ^-^, thi-1, gyrA96, relA1* | Invitrogen-Life Technologies, Inc |
| DH10b | *E. coli K12;*  *F^-^ mcrA Δ(mrr-hsdRMS-mcrBC) Φ80dlacZΔM15 ΔlacX74 endA1 recA1 deoR Δ(ara,leu)7697 araD139 galU galK nupG rpsL λ^-^* | Invitrogen-Life Technologies, Inc. |
| MG1655 | *E. coli K12; F^–^ λ^–^ilvG rfb-50 rph-1* | [28] |
| ULS153 | *Δlac* derivative of MG1655 | This study |
| ULS117 | MG1655 *speG* | This study |
| 2457T | *S. flexneri 2a* | WRAIR* |
| 2457TFd | 2457T *virF* | This study |
| M90T | *S. flexneri 5a* | [31] |
| M90TEd | M90T *speE* | This study |
| SfZM43 | *S. flexneri 6* | [30] |
| SfZM49 | *S. flexneri 3a* | [30] |
| SfZM50 | *S. flexneri 1b* | [30] |
| YSH6000 | *S. flexneri 2a* | [29] |
| SfZM53 | *S. flexneri 4* | [30] |
| SfZM46 | *S. flexneri 2a* | [30] |
| Sb411, 481, 483 | *S. boydii* C (1-7) | ISS** |
| Sb51, 484 | *S. boydii* C2 (12-15) | ISS** |
| Sb485 | *S. boydii* C3 (16-18) | ISS** |
| Sd96.29 | *S. dysenteriae A1* | ISS** |
| SdZM603 | *S. dysenteriae A1* | Somalia |
| Sd12 | *S. dysenteriae A2* | IPC*** |
| Sd16.81 | *S. dysenteriae A2* | ISS** |
| Sd4105.65 | *S. dysenteriae A2* | IPC*** |
| SsIP1,2 , 3, 4, 5, 6 | *S. sonnei* | IPC*** |
| SsZM279, 328 | *S. sonnei* | Somalia |
| Plasmids |  |  |
| pGEM-T easy | TA cloning vector | Promega inc. |
| pULS11 | pGEM-T easy derivative containing *ynfB speG* operon from MG1655 | This study |
| pULS12 | pGEM-T easy derivative containing *ynfB speG* operon from Sd12 | This study |
| pKD46 | Red recombinase expression plasmid | [56] |
| pKD13 | *kan*-containing plasmid, template for PCR | [56] |
| pRS414 | LacZ protein fusion vector | [58] |
| pULS7 | pRS414 derivative carrying P *_ynfB_* *_speG_*MG1655 | This study |
| pMYSH6504 | pBR322 derivative containing the *S. flexneri virF* gene | [29] |
| pMY6504R | pMYSH6504 derivative depleted of virF gene | This study |
| pMYSH6520 | pSC101 derivative containing the *S. flexneri virF* gene | [29] |
| pMY6520R | pMYSH6520 derivative depleted of virF gene | This study |
| pACYC184 | Low copy number cloning vector | [35] |
| pULS37 | pACYC184 derivative carrying the *ynfB* *speG* operon from MG1655 | This study |
| pULS55 | pACYC184 derivative carrying the *ynfB* gene from MG1655 | This study |
| pGIP7 | pACYC184 derivative carrying *lacI* gene and *P_lac_* region | [59] |
| pULS13 | pGIP7 derivative carrying *speG* gene from MG1655 | This study |

*WRAIR: Walter Reed Army Institute of Research. **ISS: Istituto Superiore di Sanità. ***IPC (Institut Pasteur Collection)
