# Supplementary material for: A New Piece of the Shigella Pathogenicity Puzzle: Spermidine Accumulationby Silencing of the speG Gene
Source: PLoS One. 2011 Nov 10;6(11):e27226. doi: 10.1371/journal.pone.0027226 (PMC3213128; doi:10.1371/journal.pone.0027226)
Supplement: Table S2 — E. coli genes induced by Shigella virF gene. (DOC) [file pone.0027226.s002.doc]

Table S2. E. coli genes induced by Shigella virF gene.

ID Genes	notes	Fold expression*	Conserved or eliminated in Shigella genomes	
B1868	orf, hypothetical protein; yece	33,86	eliminated	
B1354	orf, hypothetical protein; b1354	4,37	eliminated	
B1584	spermidine n1-acetyltransferase; speg	3,57	eliminated	
B1356	orf, hypothetical protein; ydar	3,35	eliminated	
B0100	orf, hypothetical protein; b0100	3,04	eliminated	
B0346	transcriptional regulator for mhp operon; mhpr	2,28	eliminated	
B0260	putative amino acid/amine transport protein; ykfd	2,23	eliminated	
B1166	orf, hypothetical protein; ymgb	2,22	eliminated	
B4176	orf, hypothetical protein; yjet	14,51	conserved	
B3687	heat shock protein; ibpa	9,34	conserved	
B4143	groel, chaperone hsp60, peptide-dependent atpase, heat shock protein; mopa	8,93	conserved	
B0473	chaperone hsp90, heat shock protein c 62,5; htpg	7,95	conserved	
B2038	dtdp-6-deoxy-d-glucose-3,5 epimerase; rfbc	6,64	conserved	
B1929	putative transport system permease protein; yede	6,51	conserved	
B1321	putative ec 2,1 enzymes; ycjx	6,00	conserved	
B3678	putative sulfatase; yidj	5,93	conserved	
B0447	putative lrp-like transcriptional regulator; ybao	5,82	conserved	
B0721	succinate dehydrogenase, cytochrome b556; sdhc	5,69	conserved	
B0014	chaperone hsp70; dna biosynthesis; autoregulated heat shock proteins; dnak	5,52	conserved	
B0439	dna-binding, atp-dependent protease la; heat shock k-protein; lon	5,46	conserved	
B2943	galactose-proton symport of transport system; galp	4,70	conserved	
B1189	d-amino acid dehydrogenase subunit; dada	4,69	conserved	
B1254	orf, hypothetical protein; ycib	4,67	conserved	
B3293	orf, hypothetical protein; yhdn	4,26	conserved	
B1180	putative isomerase; b1180	4,07	conserved	
B2615	orf, hypothetical protein; yfjb	3,98	conserved	
B1288	enoyl-[acyl-carrier-protein] reductase (nadh); fabi	3,98	conserved	
B3476	periplasmic binding protein for nickel; nika	3,96	conserved	
B0415	riboflavin synthase, beta chain; ribh	3,92	conserved	
B1878	flagellar protein; flhe	3,91	conserved	
B3222	putative nagc-like transcriptional regulator; yhci	3,83	conserved	
B2746	orf, hypothetical protein; ygbb	3,79	conserved	
B1894	is1 protein insa; insa_5	3,66	conserved	
B0959	orf, hypothetical protein; b0959	3,63	conserved	
B4245	aspartate carbamoyltransferase, catalytic subunit; pyrb	3,58	conserved	
B1779	glyceraldehyde-3-phosphate dehydrogenase a; gapa	3,49	conserved	
B0125	hypoxanthine phosphoribosyltransferase; hpt	3,48	conserved	
B3356	orf, hypothetical protein; yhfa	3,41	conserved	
B3569	putative regulator of xyl operon; xylr	3,37	conserved	
B2234	ribonucleoside diphosphate reductase 1, alpha subunit, b1; nrda	3,37	conserved	
B2029	gluconate-6-phosphate dehydrogenase, decarboxylating; gnd	3,34	conserved	
B0876	orf, hypothetical protein; ybjd	3,31	conserved	
B0086	d-alanine:d-alanine-adding enzyme; murf	3,21	conserved	
B1913	excinuclease abc, subunit c; repair of uv damage to dna; uvrc	3,20	conserved	
B3789	glucose-1-phosphate thymidylyltransferase; rffh	3,16	conserved	
B2891	peptide chain release factor rf-2; prfb	3,11	conserved	
B2713	involved in electron transport from formate to hydrogen, fe-s centers; hydn	3,11	conserved	
B2557	phosphoribosylformyl-glycineamide synthetase = fgam synthetase; purl	3,11	conserved	
B0151	atp-binding component of hydroxymate-dependent iron transport; fhuc	3,06	conserved	
B2678	high-affinity transport system for glycine betaine and proline; prow	3,03	conserved	
B0643	putative alpha helical protein; ybel	3,02	conserved	
B0762	orf, hypothetical protein; b0762	3,02	conserved	
B1848	orf, hypothetical protein; yebg	3,00	conserved	
B3067	rna polymerase, sigma(70) factor; regulation of proteins induced at high temperatures; rpod	2,98	conserved	
B2024	n-(5'-phospho-l-ribosyl-formimino)-5-amino-1-(5'- phosphoribosyl)-4-imidazolecarboxamide isomerase; hisa	2,97	conserved	
B0420	1-deoxyxylulose-5-phosphate synthase; flavoprotein; dxs	2,96	conserved	
B1244	oligopeptide transport permease protein; oppb	2,93	conserved	
B0953	ribosome modulation factor; rmf	2,93	conserved	
B1825	orf, hypothetical protein; b1825	2,92	conserved	
B3959	acetylglutamate kinase; argb	2,90	conserved	
B4194	orf, hypothetical protein; sgab	2,89	conserved	
B0698	atpase of high-affinity potassium transport system, a chain; kdpa	2,87	conserved	
B3382	orf, hypothetical protein; yhfy	2,86	conserved	
B4147	elongation factor p (ef-p); efp	2,83	conserved	
B3932	heat shock protein hslvu, proteasome-related peptidase subunit; hslv	2,83	conserved	
B2568	leader peptidase (signal peptidase i); lepb	2,80	conserved	
B1656	superoxide dismutase, iron; sodb	2,79	conserved	
B3616	threonine dehydrogenase; tdh	2,75	conserved	
B1236	glucose-1-phosphate uridylyltransferase; galu	2,75	conserved	
B1216	sodium-calcium/proton antiporter; chaa	2,75	conserved	
B4222	orf, hypothetical protein; ytfp	2,74	conserved	
B0802	orf, hypothetical protein; ybij	2,70	conserved	
B1702	phosphoenolpyruvate synthase; ppsa	2,65	conserved	
B3641	putative transcriptional regulator; ttk	2,65	conserved	
B1208	orf, hypothetical protein; ychb	2,64	conserved	
B0211	transcriptional regulator for nitrite reductase (cytochrome c552); dnir	2,63	conserved	
B0928	aspartate aminotransferase; aspc	2,61	conserved	
B0466	orf, hypothetical protein; ybam	2,59	conserved	
B1558	cold shock protein; cspf	2,58	conserved	
B0658	putative transport protein; ybex	2,58	conserved	
B4052	replicative dna helicase; part of primosome; dnab	2,57	conserved	
B3122	orf, hypothetical protein; b3122	2,57	conserved	
B3704	rnase p, protein component; protein c5; processes trna, 4,5s rna; rnpa	2,57	conserved	
B0114	pyruvate dehydrogenase (decarboxylase component); acee	2,55	conserved	
B3449	glycerophosphodiester phosphodiesterase, cytosolic; ugpq	2,54	conserved	
B0991	suppresses faba and ts growth mutation; sfa	2,52	conserved	
B4395	phosphoglyceromutase 2; gpmb	2,52	conserved	
B4173	gtp - binding subunit of protease specific for phage lambda cii repressor; hflx	2,51	conserved	
B2908	proline aminopeptidase p ii; pepp	2,51	conserved	
B0134	3-methyl-2-oxobutanoate hydroxymethyltransferase; panb	2,50	conserved	
B0104	gmp reductase; guac	2,50	conserved	
B0850	orf, hypothetical protein; ybjc	2,50	conserved	
B0416	transcription termination; l factor; nusb	2,49	conserved	
B2688	gamma-glutamate-cysteine ligase; gsha	2,49	conserved	
B3399	putative phosphatase; yrfg	2,48	conserved	
B1268	orf, hypothetical protein; yciq	2,48	conserved	
B0967	putative oxidoreductase; b0967	2,47	conserved	
B2521	putative thiosulfate sulfurtransferase; ssea	2,47	conserved	
B0092	d-alanine-d-alanine ligase b, affects cell division; ddlb	2,44	conserved	
B0852	ribosomal protein s6 modification protein; rimk	2,43	conserved	
B0641	a minor lipoprotein; rlpb	2,41	conserved	
B0392	orf, hypothetical protein; b0392	2,40	conserved	
B1415	aldehyde dehydrogenase, nad-linked; alda	2,40	conserved	
B2614	phage lambda replication; host dna synthesis; heat shock protein; protein repair; grpe	2,40	conserved	
B1583	orf, hypothetical protein; b1583	2,37	conserved	
B0617	citrate lyase acyl carrier protein (gamma chain); citd	2,37	conserved	
B2962	orf, hypothetical protein; yggx	2,37	conserved	
B1496	putative atp-binding component of a transport system; ydda	2,36	conserved	
B3066	dna biosynthesis; dna primase; dnag	2,35	conserved	
B2305	orf, hypothetical protein; yfci	2,33	conserved	
B0927	orf, hypothetical protein; ycbl	2,31	conserved	
B3878	putative glycosidase; yihq	2,31	conserved	
B3178	degrades sigma32, integral membrane peptidase, cell division protein; hflb	2,28	conserved	
B2154	putative esterase (ec 3,1,1,-); yeig	2,26	conserved	
B2711	putative oxidoreductase; ygbd	2,24	conserved	
B0264	is1 protein insb; insb_2	2,24	conserved	
B0313	probably transcriptional repressor of bet genes; beti	2,23	conserved	
B2223	short chain fatty acid transporter; atoe	2,20	conserved	
B2252	protein induced by aluminum; ais	2,20	conserved	
B3982	component in transcription antitermination; nusg	2,16	conserved	
B3400	orf, hypothetical protein; yrfh	2,16	conserved	
B3743	regulator for asna, asnc and gida; asnc	2,16	conserved	
B1795	orf, hypothetical protein; yeaq	2,16	conserved	
B3487	putative membrane protein; yhii	2,15	conserved	
B3041	3,4 dihydroxy-2-butanone-4-phosphate synthase; ribb	2,13	conserved	
B0760	atp-binding component of molybdate transport system; modf	2,08	conserved	
B3751	d-ribose periplasmic binding protein; rbsb	2,07	conserved	
B0525	peptidyl-prolyl cis-trans isomerase b (rotamase b); ppib	2,04	conserved	
B0178	histone-like protein, located in outer membrane or nucleoid; hlpa	2,03	conserved	

*Expression has been calculated as fold ratio of MG1655 pMYSH6504 vs MG1655 pMY6504R
