# Supplementary material for: A New Piece of the Shigella Pathogenicity Puzzle: Spermidine Accumulationby Silencing of the speG Gene
Source: PLoS One. 2011 Nov 10;6(11):e27226. doi: 10.1371/journal.pone.0027226 (PMC3213128; doi:10.1371/journal.pone.0027226)
Supplement: Table S3 — Oligos used in this study. (DOC) [file pone.0027226.s003.doc]

Table S3. Oligos used in this study

| **oligos** | **Sequence 5’-3’** |
| --- | --- |
| *def* | GATTGTAATAACGGCATTTCAGGCCAGAGGCGAGAAATTCCGGGGGATCCGTCGACC |
| *der* | GGCATGAAACGCTACACGACCAGTTTGGGCAGTACTGTGTAGGCTGGAGCTGCTTC |
| *dff* | ATGCAAAAAGGTGTTCAATGACGGTTAGCTCAGGCAATGAGTGTAGGCTGGAGCTGCTTC |
| *dfr* | GACGCCATCTCTTCTCGATGTTTTTTTCAACAATCTTCCATTCCGGGGATCCGTCGACC |
| *dgf* | GAAGCCTTTGTTTGAACTCTCTGATCTGTATGATAAGCGTGTAGGCTGGAGCTGCTTCG |
| *dgr* | ATAGAGATTGAGAACGGTAAAGCCATAGTCCATTGCATTCCGGGGATCCGTCGACC |
| *dlf* | GCAGGAAACGCCAATAACATACAGTGACAAAAACCATGTGTAGGCTGGAGCTGCTTCG |
| *dlr* | CTGGCTGGCTGGCATAAATATCTCACTCGCAATCAAATATTCCGGGGATCCGTCGACC |
| *ect* | ATGCCAAGCGCCCACAGTGTT |
| *gof* | GGAGGATCCATGCCAAGCGCCCAC |
| *gor* | TATGGATCCTATTGTGCGGTCGGCT |
| *kgf* | CCTCGCTGGCGGATATCAT |
| *kgr* | GGCGCTTGCGGCTTT |
| *mdf* | CCTGGTACAGCAAGTTGCG |
| *mdr* | CGTAGGCACATTCGACAACG |
| *pgf* | NGAATTCCCACAACCAGGGCAGAAAGCA |
| *pgr* | GGATCCTCAAACCAGTAACGCATCAC |
| *rgf* | AACGCCAGTGTGATGCGTTA |
| *rgr* | CAGAGAGTTCAACAAAGGCTTCGT |
| *sbt* | GCACCAACATAAACCAAGGGAGAA |
| *sft* | TGCCAAGCGCCCACAGTTAA |
| *ygt* | GAAGGAGTAAAACCCGCCGTC |
